# Quantification of the Dynamics of the Vascular Flows in the Cerebral Arterial and Venous Trees

**DOI:** 10.3390/biomedicines13051106

**Published:** 2025-05-01

**Authors:** Heimiri Monnier, Kimi Owashi, Pan Liu, Serge Metanbou, Cyrille Capel, Olivier Balédent

**Affiliations:** 1 CHIMERE UR 7516, Jules Verne University of Picardy, 80000 Amiens, France; 2 Medical Image Processing Department, CHU Amiens-Picardie University Hospital, 80000 Amiens, France; 3 Radiology Department, CHU Amiens-Picardie University Hospital, 80000 Amiens, France; 4 Neurosurgery Department, CHU Amiens-Picardie University Hospital, 80000 Amiens, France

**Keywords:** phase-contrast MRI, blood flow quantification, cerebral vascularization, venous heterogeneity, alternative pathway

## Abstract

**Objective:** Cerebral vascularization is made of the symmetrical arterial system, with muscular walls, and the venous system, more variable and dominated by sinuses and jugular veins. Factors like age and posture influence this network, complicating its study. Phase-contrast MRI is the gold standard for quantifying cerebral circulation. This study aimed to quantify the dynamics of the cerebral blood system using PC-MRI. **Materials and Methods:** Thirty-six healthy adults participated. Imaging was performed on a 3T MRI (Philips Achieva) in a supine position. Two slices were acquired: intracranial and extracranial. In-house software analyzed flow curves over a cardiac cycle. Each vessel’s contribution was evaluated. **Results:** Extracranial venous drainage was categorized as jugular-dominant, equivalent, or peripheral-dominant. A similar classification applied intracranially. Intracranial flows showed low variability (5–9%), while extracranial venous flows, especially in the internal jugular veins, had higher variability (17–21%). Some extracranial veins were absent. **Conclusions:** There is significant venous heterogeneity in the extracranial region. PC-MRI enables the quantification of cerebral dynamics.

## 1. Introduction

**Figure 1:**
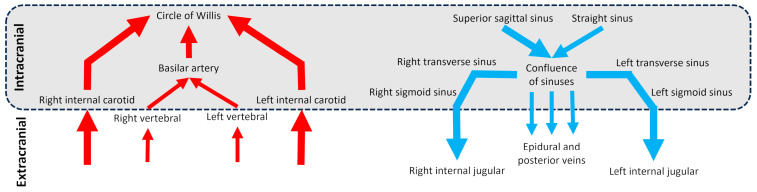
Diagram of the cerebral arterial and venous trees in intracranial and extracranial regions. The schematic vessels represent the main vessels studied for brain vascularization.

Cerebral vascularization depends on two distinct but interconnected systems: the arterial system and the venous system (Figure 1), each with unique anatomical features. At the extracranial level, the brain is primarily supplied by four main arteries: the right and left internal carotid arteries and the right and left vertebral arteries. Intracranially, the internal carotid arteries provide the majority of cerebral blood flow, entering the skull to supply the anterior and middle parts of the brain. The vertebral arteries ascend on either side of the spine and merge inside the cranial cavity to form the basilar artery in front of the circle of Willis, a critical arterial junction [1]. This symmetrical arterial pattern is well-established and shows remarkable anatomical consistency between individuals [2]. Venous drainage of the brain occurs primarily through the dural venous sinuses, derived from folds of the dura mater [3]. The superior sagittal sinus drains superficial cortical areas, while the straight sinus handles deep drainage. These converge at the confluence of sinuses continuing through the transverse and sigmoid sinuses and exiting the skull via the jugular foramen to become the internal jugular veins. A secondary venous system composed of epidural and posterior cervical veins, deep cervical veins, also contributes to drainage [4]. This secondary network is less well-known, more variable between individuals, asymmetrical, and dependent on body position [5,6]. Although often underemphasized, the venous compartment constitutes the majority of intracranial blood volume, with only about 30% of this volume located in the arterial system [7]. This makes the venous system not only a passive conduit for outflow, but a key player in cerebral hemodynamics. Its high compliance allows it to absorb fluctuations in blood volume and ICP, serving as a dynamic buffer to protect brain homeostasis.

The cerebral arterial system maintains high pressure, around 100 mmHg, and high pulsatility, owing to the muscular walls of arteries. Blood flow is synchronized with the cardiac cycle: during systole, arterial inflow increases due to the heart’s contraction; during diastole, flow decreases as the heart refills. This dynamic results in cyclic fluctuations of cerebral blood volume, which are regulated to preserve stable intracranial pressure (ICP) [8]. In contrast, the cerebral venous system operates under lower pressure and shows greater anatomical and functional variability. It plays a crucial role in adapting to changes in body posture and gravity. In the supine position, the internal jugular veins are the primary drainage pathways. However, when standing, these veins are subject to negative pressure, causing them to collapse and redirect drainage to the vertebral venous plexus, an alternative drainage system [5,6,9]. Unlike the cerebral arterial system, which is relatively consistent across individuals, the cerebral venous system shows great heterogeneity, making anatomical systematization difficult [10,11]. Intracranially, venous drainage exhibits significant variations at the confluence of sinuses, with some cases even showing a complete absence [12]. According to Saiki et al. [13], drainage by the right transverse sinus is generally predominant, and the right internal jugular vein is usually larger than the left one. The venous network also shows significant heterogeneity at the cervical level [14]. Among the factors that can influence this variability, aging affects these structures as well, leading to a reduction in cerebral blood flow with age [15]. Moreover, Ciuti et al. [16] demonstrated that blood flow in the internal jugular and vertebral veins is significantly reduced when transitioning from a supine to a sitting position, highlighting the importance of considering these alternative drainage pathways in clinical assessment. The anatomical and functional features have important clinical implications, especially in understanding cerebral fluid dynamics and conditions involving PIC dysregulation.

Since the late 1980s, Magnetic Resonance Imaging (MRI) has revolutionized the study of blood flow with the introduction of the CINE Phase Contrast (CINE-PC) technique [17,18,19]. This sequence synchronizes image acquisition with the cardiac cycle, providing a precise temporal blood flow analysis. This advancement not only enables visualization of blood vessels but also allows for accurate quantification of flow rates. The first applications of the CINE-PC sequence made it possible to explore both the vertebral arteries and internal carotid arteries [17,19]. Today, PC-MRI is the gold standard for the quantification of cerebral fluids dynamics, especially in the exploration of the intracranial vascular system. Unlike Doppler ultrasound, which cannot pass through the skull, PC-MRI offers a non-invasive method for studying blood flow in the brain, allowing for a detailed analysis of intracranial flows. Measurements taken using PC-MRI reveal distinct characteristics of cerebral arterial and venous flow. Bhadelia et al. showed in young adults that the flow in the internal carotid artery presented a peak velocity that can reach 60 cm/s, while venous flow in the internal jugular vein was slower, with a peak around 47 cm/s [20,21]. It is assumed that the average cerebral arterial inflow equals the average cerebral venous outflow over a cardiac cycle. However, the average flow in the internal jugular veins is highly variable and does not average alone the total incoming arterial flow. This difference in flow needs to be compensated for by the contribution of a cerebral peripheral plexus veins system [14].

Blood flow in the cerebral vessels is closely related to the heart’s pulsatile action, resulting in variations in flow rate throughout the cardiac cycle. During systole, the rapid contraction of the heart generates a peak velocity in arterial blood flow, reflecting the sudden influx of blood into the arteries. This phase of high pressure leads to an increase in cerebral blood volume, triggering compensatory mechanisms to maintain a stable and normal mean intracranial pressure (ICP) around 10 to 15 mmHg in healthy adults [22]. During diastole, the heart relaxes and fills with blood, causing a gradual decrease in arterial blood flow. The dynamics of cerebral blood flow differ significantly between the arterial and venous systems. During systole, the inflow of arterial blood into the skull increases cerebral blood volume. This increase, in accordance with the Monro–Kellie principle [7], is compensated for by the oscillation of cerebrospinal fluid (CSF) toward the spinal compartment, thus maintaining ICP balance. During diastole, venous blood exits the skull to return to the heart, reducing cerebral blood volume. In response, CSF returns to the cranial cavity, restoring the balance between arterial inflow and venous outflow [23]. This balance is essential for maintaining a stable and normal mean ICP. ICP also varies based on body position. In the supine position, ICP is naturally higher. When transitioning to a seated position, ICP decreases non-linearly [24]. Initially, this decrease is rapid as the internal jugular veins are not yet compressed, allowing efficient venous blood drainage. Once the jugular veins collapse in the upright position, the decrease in ICP becomes slower [24]. Any disruption of this delicate balance between arterial inflow, venous outflow, and CSF movement can impair ICP regulation and potentially lead to pathologies.

The distribution of cerebral arterial flow among the various branches of the cerebral venous tree remains poorly understood. While arterial dynamics are relatively well characterized, how arterial blood flow is distributed and subsequently drained by the cerebral venous system remains largely unexplored. This lack of knowledge is clinically relevant, as understanding the variability of venous drainage is critical for improving the diagnosis and management of cerebrovascular pathologies. The objective of this study is to quantify the distribution of arterial flow within the various branches of the cerebral venous tree in a control population using PC-MRI. By providing new insights into cerebral venous dynamics and interindividual variability, this study may contribute to a better understanding of how venous heterogeneity influences ICP regulation and may help identify early markers of venous-related disorders.

## 2. Materials and Methods

### 2.1. Study Population

This study included a population of 36 adult volunteers, consisting of 20 men and 16 women, with a mean age of 25 ± 4 years and a mean heart rate of 72 ± 12 beats per minute (BPM). Participants were selected based on specific inclusion criteria, excluding individuals with contraindications to MRI, known neurological or vascular conditions, or any abnormalities detected during the preliminary clinical examination. This study was approved by an independent ethics committee (CPP Nord Ouest II, Amiens, France, reference: PI2019_843_0056) and conducted in accordance with the Declaration of Helsinki. Each examination lasted approximately 30 min and was preceded by an interview to assess eligibility for participation, followed by the reading and signing of an information and consent form.

### 2.2. Data Acquisition

We used a 3T clinical research MRI (Philips Achieva, Eindhoven, The Netherlands) with a maximum gradient of 80 mT/m. Acquisitions were performed using a 32-channel head coil. Participants were examined in a supine position.

**Figure 2:**
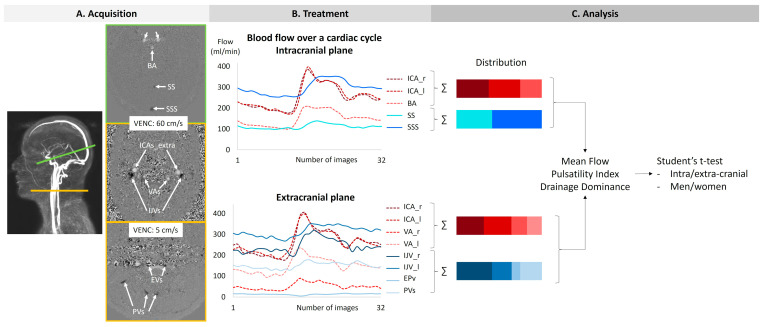
The procedure of acquisition, treatment, and analysis. The green line on the angiography represents the intracranial section, and the yellow line represents the extracranial section. ICAs_intra: internal carotid arteries at the intracranial level. BA: basilar artery. SS: straight sinus. SSS: superior sagittal sinus. ICAs_extra: internal carotid arteries at the extracranial level. Vas: vertebral arteries. IJVs: internal jugular veins. Evs: epidural veins. PVs: posterior veins.

**Figure 3:**
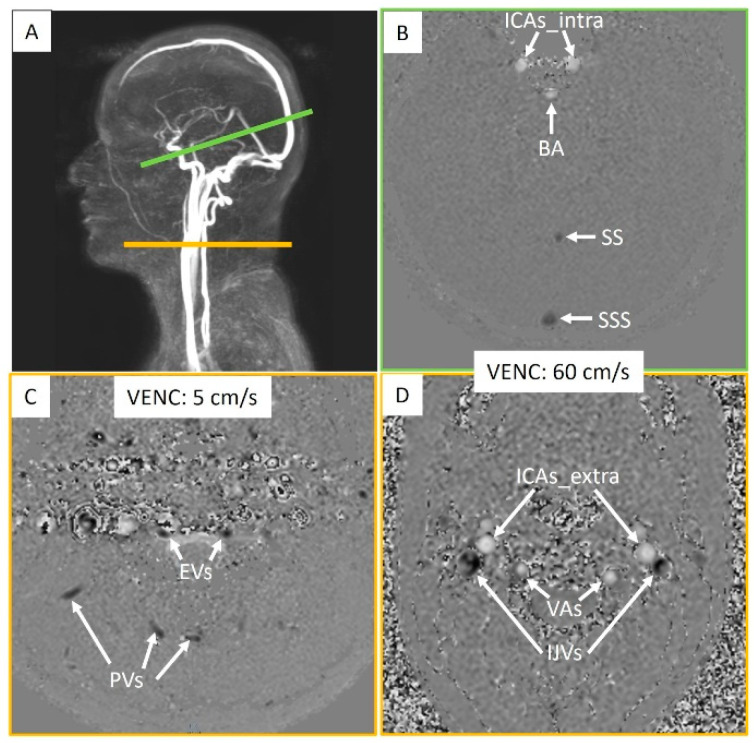
Intracranial and extracranial acquisition planes for blood flow measurements. (**A**) Individual angiography to determine the slice planes. The green line represents the intracranial section, and the yellow line represents the extracranial section. (**B**) Intracranial plane including the internal carotid arteries (ICAs_intra), basilar artery (BA), straight sinus (SS), and superior sagittal sinus (SSS). (**C**) Extracranial plane including the epidural veins (EVs) and posterior veins (PVs). (**D**) Extracranial plane including the internal carotid arteries (ICAs_extra), vertebral arteries (VAs), and internal jugular veins (IJVs). Two velocity encoding (VENC) settings were used extracranially to accommodate smaller venous flows. Each vessel’s flow was quantified.

Two phase-contrast acquisition planes were individually localized on a 3D angiography (Figure 2A). The extracranial plane included the right and left internal carotid arteries, the right and left vertebral arteries, the right and left internal jugular veins, the right and left epidural veins, and the small posterior veins (Figure 3). Due to variations in flow velocities among the studied vessels, the extracranial plane was acquired twice with different velocity encoding (Venc) settings: 60 cm/s or 120 cm/s (in cases of aliasing) and 5 cm/s or 20 cm/s (in cases of aliasing). The intracranial plane included the right and left internal carotid arteries, the basilar artery, the straight sinus, and the superior sagittal sinus (Figure 3). Blood flow directed towards the heart was defined as positive and appears as black pixels on the phase images.

The CINE-CP sequence required a plethysmograph to achieve cardiac synchronization. Each cycle produced 32 images, enabling the reconstruction of average flow dynamics over a cardiac cycle.

The main parameters of the sequences used are detailed in Table 1.

### 2.3. Image Processing

Based on the acquired PC-MRI images, a semi-automatic fixed segmentation algorithm was used to identify vessel region of interest (ROI). This method analyzes the velocity spectrum of each pixel and determines whether it belongs to a vessel based on the amplitude of the cardiac frequency component [23]. A fully automated background field correction was then applied to eliminate velocity offsets. This was achieved by identifying static tissue surrounding the vessel region using both magnitude and velocity images [25] and setting its average velocity as the new zero-velocity reference. In addition, the software corrected phase aliasing when the blood velocity exceeded the predefined Venc. Finally, the software calculated flow rate curves by multiplying the average velocity within the vessel ROI by its area for each of the 32 images (Figure 2B).

The accuracy and reproducibility of this software have been assessed in previous studies [23,26]. Their findings support the robustness of the software for the quantification of vascular blood flow dynamics from 2D CINE PCMRI. Reproducibility of the software to segment vascular vessels was judged acceptable and presented less than 5% difference for the flow rate measurements between different operators and less than 10% of difference with a known flow rate generated by a flow pump.

**Figure 4:**
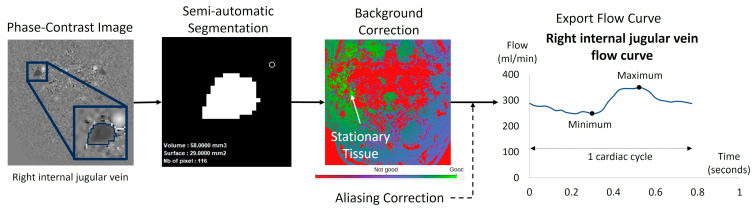
Example of flow quantification for a right internal jugular vein using Flow software. The surface of the segmented region of interest (ROI) was semi-automatically obtained using in-house software dedicated to blood flow analysis. The ROI surface was multiplied by the mean velocities of the 32 acquisition time points of the cardiac cycle. The software applied a background correction using the surrounding stationary tissue to avoid eddy current effects. Finally, the software provided the ROI curve flow dynamic over the 32 temporal points of the cardiac cycle. We applied in all the dynamic curves a manual visual check to verify no velocity aliasing occurred. In cases of blood velocity higher than the Venc but no more than 2 Venc, the software can correct this velocity aliasing error as previously described [23].

For each flow curve over the cardiac cycle of each vessel, the minimum, maximum, and average values were automatically calculated to determine the pulsatility index (Figure 4).

### 2.4. Calculation of the Pulsatility Index

The pulsatility index of each vessel was calculated as follows (Figure 3):

$$\mathrm{Pulsatility}\;\mathrm{index}=\frac{Maximum\;flow-Minimum\;flow}{Mean\;flow}$$

### 2.5. Cerebral Flows

Extracranial arterial flow (ExtraArt) was the sum of the flows from the right and left internal carotid arteries, as well as those of the right and left vertebral arteries. These measurements were taken at the C2–C3 cervical level, outside the skull (Figure 3). Extracranial venous flow (ExtraVein) was determined by the sum of the flows of the right and left internal jugular veins, identified on images acquired with a Venc of 60 cm/s. Additionally, ExtraVein flow included the flows from the right and left epidural veins, as well as peripheral veins, identified on images acquired with a Venc of 5 cm/s, also at the extracranial cervical level (Figure 3).

Intracranial arterial flow (IntraArt) was obtained by summing the flows of the right and left internal carotid arteries, as well as that of the basilar artery at the intracranial level (Figure 3). Intracranial venous flow (IntraVein) was calculated from the sum of the flows of the straight sinus and the superior sagittal sinus, measured at the intracranial level (Figure 3).

### 2.6. Distribution

The average value of IntraArt represented the total arterial flow entering the brain at the intracranial level. Similarly, the average value of IntraVein determined the total flow exiting the brain at the intracranial level.

These average flow values were then used to evaluate the contribution of each vessel relative to the overall flow, either arterial or venous. A ratio was calculated for each vessel, representing its relative share in the total intracranial flow, whether arterial or venous (Figure 2C).

The same calculation method was applied to the flows measured at the extracranial level. Average values of ExtraArt and ExtraVein were calculated accordingly.

### 2.7. Cerebral Drainage Dominance

If the flow through the internal jugular veins exceeded 60% of the ExtraArt, the extracranial venous drainage was considered dominated by the internal jugular veins. If the flow through the internal jugular veins accounted for 40% to 60% of the ExtraArt, then the extracranial venous drainage was considered shared, involving both the jugular veins and the peripheral venous network. Finally, if the flow through the internal jugular veins represented less than 40% of the ExtraArt, then the peripheral drainage became dominant, indicating reliance on the peripheral network for extracranial drainage.

Similarly, if the combined flow of the straight and superior sagittal sinuses represented more than 60% of the IntraArt, the intracranial venous drainage was classified as dominated by these sinuses. If the flow of the sinuses accounted for 40% to 60% of IntraArt, the intracranial drainage was then classified as mixed, indicating balanced participation of the sinuses and other venous pathways. If the flow of the sinuses represented less than 40% of the IntraArt, peripheral drainage was considered dominant, meaning that other venous pathways accounted for the majority of the intracranial drainage.

### 2.8. Data Analysis

Comparisons of extracranial and intracranial arterial and venous flows were conducted between men and women. For each sex, arterial and venous flows were analyzed at both the extracranial and intracranial levels and then compared to each other. Additionally, the pulsatility index of arterial vessels was compared between men and women. As the data followed a normal distribution, all statistical analyses were performed using a Student’s *t*-test. No correction for multiple comparisons was applied, as the analyses were exploratory and each comparison was hypothesis-driven and biologically justified.

## 3. Results

**Figure 5:**
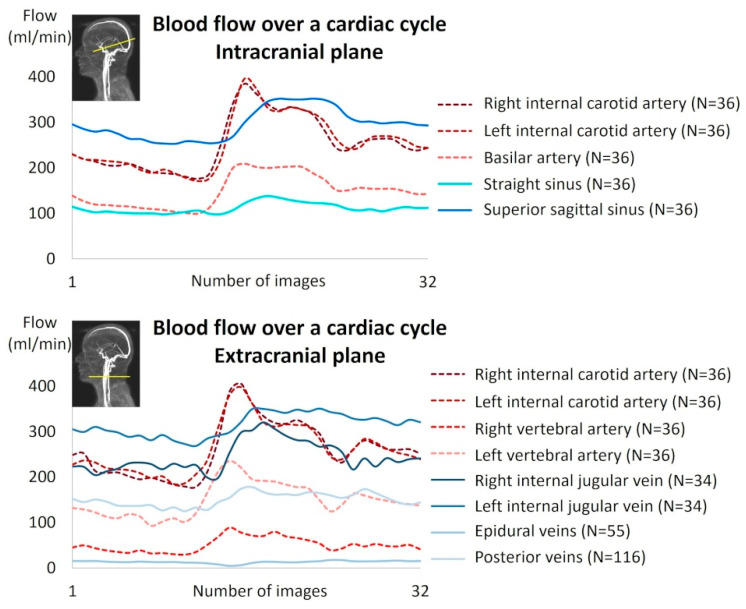
Example of cerebral blood flow reconstruction over a cardiac cycle for a subject.

In the intracranial region, all vessels were identifiable and quantifiable. In the extracranial region, all arteries were identifiable and quantifiable, whereas some veins were absent or presented no flow in several subjects due to the variability of cerebral venous drainage anatomy in humans (Table 2). Figure 5 shows an example of artery and vein quantification for a subject over a cardiac cycle in the intracranial and extracranial planes. The good quality of the images was assessed by an experienced neuroradiologist and a biophysicist.

**Figure 6:**
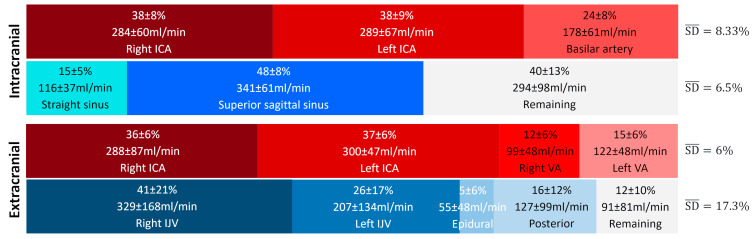
Average proportion of arterial and venous flows at the intracranial and extracranial levels. The $\bar{SD}$ represents the average variability of the main vessels and provides a summarized view of the variability within the given vascular tree structure and plane. High variability in venous contribution is observed at the extracranial level, with an $\bar{SD}$ value more than twice as large as the others. ICA: internal carotid artery, VA: vertebral artery, IJV: internal jugular vein.

**Figure 7:**
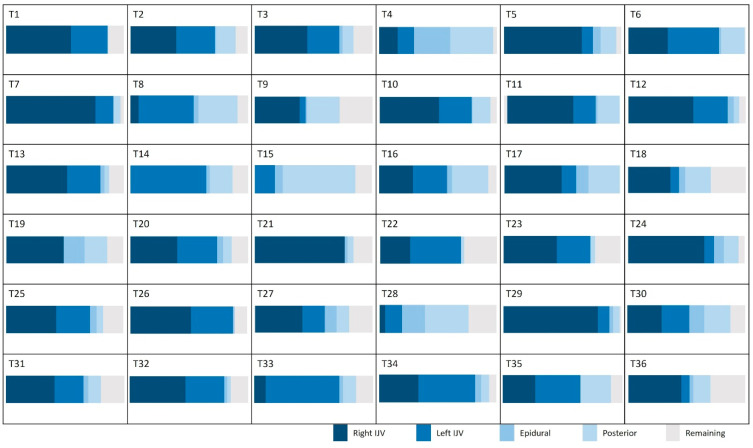
Proportion of venous flow in the extracranial plane for the studied population. IJV: internal jugular vein.

Figure 6 presents the average contributions of the vessels studied at the intracranial and extracranial levels. An interindividual homogeneity is observed at the intracranial level, with standard deviations ranging from 5% to 9%. At the extracranial level, a high venous variability is noted, particularly in the contribution of the internal jugular veins, which exhibit an average standard deviation of 17.3%. When examining the venous proportions at the extracranial level for each participant (Figure 7), a high variability between individuals is observed.

No significant differences were observed in the average flow rates between sexes in either the intracranial or extracranial planes, for both arterial and venous flows (Table 3). Similarly, no significant sex-based differences were found in the pulsatility index at the intracranial plane (arterial and venous), or the extracranial plane (venous) (Table 3). However, the arterial pulsatility index at the extracranial plane was significantly higher in men (*p* = 0.02) (Table 3).

The average venous flow rate was significantly higher at the extracranial plane than at the intracranial plane for both men and women (*p* = 2 × 10^−9^ for men and *p* = 2 × 10^−8^ for women). The arterial pulsatility index was significantly higher at the extracranial plane compared to the intracranial plane for men (*p* = 4 × 10^−3^) (Table 4). The venous pulsatility index was significantly higher at the extracranial plane than at the intracranial plane for both men and women (*p* = 3 × 10^−6^ for men and *p* = 2 × 10^−6^ for women) (Table 4).

**Figure 8:**
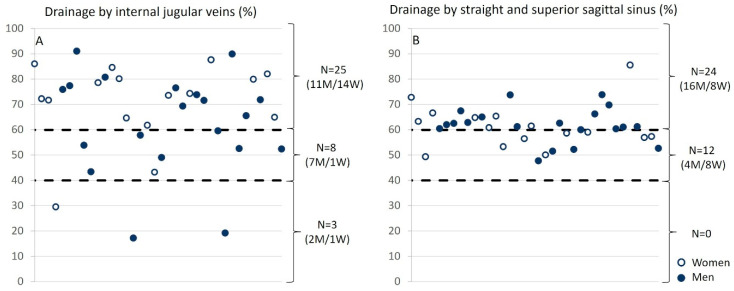
Relative drainage of the internal jugular veins and sinuses as a function of arterial input. (**A**) For internal jugular veins, three groups were identified: (i) the internal jugular veins provide the majority of drainage, meaning that the drainage from the jugulars accounts for more than 60% of the total drainage (N = 25); (ii) the posterior venous system dominates (N = 3); (iii) a balance between the different veins (N = 8) is observed. (**B**) For the sinuses, they account for more than 60% of the total drainage in 24 subjects and between 40% and 60% in 12 subjects. The empty circles represent women, and the filled circles represent men.

The analysis of relative drainage highlights distinct patterns for the internal jugular veins and sinuses in relation to arterial input. For the internal jugular veins, three groups emerge: in the majority of cases (N = 25), the internal jugular veins are the primary drainage pathway, contributing to over 60% of the total venous outflow. In contrast, a posterior venous system dominance is observed in a smaller group (N = 3). Additionally, a balanced distribution among different venous pathways is noted in eight participants. Regarding the sinuses, their drainage contributes more than 60% of the total in 24 subjects, while in 12 participants, it ranges between 40% and 60%. We did not observe any significant difference between the venous drainage of men and that of women at either level (Figure 8).

## 4. Discussion

### 4.1. Acquisition

Phase-contrast MRI is a unique technique to quantify blood flow velocities in the arterial and venous vessels intra- and extracranially. By the late 1990s, numerous publications had shown its accuracy and reproducibility in measuring blood flows [27,28,29]. To optimize sensitivity, it is essential to adjust the Venc of the MRI sequence according to the characteristics of blood flow. The internal carotid arteries, vertebral arteries, and internal jugular veins generally present faster velocities compared to smaller veins, such as epidural veins. Therefore, to maximize venous inclusion, we performed dual acquisitions at the cervical level with two different VENCs, one at 60 cm/s for larger vessels and another at 5 cm/s for the epidural and small peripheral vein flows (Figure 3).

Nevertheless, some subjects may exhibit higher flow velocities than the set VENC, resulting in velocity aliasing in the images. In cases of simple aliasing, our post-processing software can correct the issue [23]. However, if the aliasing is double, the acquisition must be repeated with a double VENC.

The advantage of PC-MRI is that arterial flows and the large jugular vein flows at the cervical level are acquired simultaneously on the same slice at the same time. Similarly, for intracranial flows, a single slice is able to capture the flows of the relevant vessels. This approach allows for a coherent and simultaneous assessment of arterial and venous flows, thus ensuring accurate analysis of hemodynamic dynamics in these two compartments.

Another advantage, compared to Doppler echography, is that PC-MRI does not involve contact with the skin, avoiding vein compression, and is not limited by the bony barrier of the skull. The main limitation of PC-MRI is that it is not an instantaneous measurement but needs cardiac synchronization to accumulate many cardiac cycle signals to ultimately reconstruct only one mean dynamic flow curve for one cardiac cycle. This requires a stable cardiac cycle frequency on the subject, and breathing effects on the flow cannot be evaluated.

As a 2D sequence, the entire vascular tree cannot be acquired in the same acquisition, necessitating a 3D phase-contrast angiography (3D PCA) to properly position the PC-MRI slices perpendicular to both arterial and venous vessels within the same slice. The advantage of 2D PC-MRI lies in its high spatial resolution and relatively short acquisition time. In the past few years, 4D PCMRI and real-time PCMRI have emerged for studying cerebral flows [30,31].

Some veins could not be identified during post-processing (Table 2). However, the fact that a vein is not identifiable in flow images does not necessarily indicate that the vein is non-existent. It is possible that the vein is not being used for venous return under the acquisition conditions (in this case, in a supine position). Additionally, the caliber of the vein or the blood flow within it may be too low to be detected by the acquisition protocol. Thus, the non-identification of a vein in PC-MRI should not be interpreted as an absence of that vein, but rather as a technical limitation related to the acquisition method and the physiological parameters of the patient at the time of the examination. To address this limitation, for each subject, we calculated the missing “remaining” venous flow (Figure 7) necessary to balance the cerebral arterial flow, based on the hypothesis that all the arterial inflow to the cranium must be drained by venous outflow at the end of the cardiac cycle.

The semi-automatic segmentation generates a fixed region of interest of the vessels for the 32 images of the cardiac cycle. Consequently, potential movement of the vessels and the variations of the caliber of the vessel during the cardiac cycle were not taken into account.

### 4.2. The System Can Also Be Studied in 4D

The assessment of cerebral hemodynamics can be performed using a 4D Flow sequence, which allows dynamic acquisition of the entire volume, providing a comprehensive view of the blood flow [32]. However, 4D Flow is less precise for flow quantification due to its lower temporal resolution compared to CINE-PC, making it less suitable for capturing rapid flow peaks. Additionally, the longer acquisition time can be problematic in the event of individual movement or other artifacts or different blood flow velocities in the different vessels. At the end, to quantify blood flow in a vessel, as we have done in the present work, 4D flow needs post-processing to go back to a single 2D slice from the volume. To make a compromise with the 4D flow, we added to our protocol a 3D PCMRI with Venc equal to 30 cm/s to obtain in less than 3 min an arterial and venous cerebral angiography.

Our protocol has several significant advantages. It provides a non-injected vascular cerebral anatomy to highlight the main vessels to quantify. The quantitative 2D CINE PCMRI acquisition takes only a maximum of 2 min per level, allowing for repeat examinations when necessary, such as in cases of movement or inappropriate Venc. With excellent temporal resolution, our method captures brief peak flows, providing higher accuracy for flow quantification.

However, both methods share a common limitation: dependence on cardiac synchronization, which excludes the influence of respiration on blood flow dynamics. To overcome this limitation, real-time PC-MRI could be a promising alternative. This method combines rapid Echo Planar Imaging (EPI) with phase contrast to generate velocity maps in less than 0.1 s continuously. It does not require cardiac synchronization, enabling the capture of continuous dynamics and low-frequency respiratory influences [33,34,35].

### 4.3. Influence of Respiration

Using EPI-CP, the evaluation of arteriovenous parameters allows for the observation of the effects of free breathing on blood flow [34,35]. After 3 min of hyperventilation, a significant decrease in flow velocity in the cerebral veins is observed [36]. This phenomenon is attributed to variations in intracranial and thoracic pressures caused by breathing, which directly influence venous flow.

The influence of respiration on venous flow has also been confirmed by Doppler measurements. It has been shown that flow variations in the jugular veins are correlated with respiratory cycles [37]. Indeed, during inspiration, an increase in intrathoracic pressure occurs, which causes a decrease in venous return, while expiration promotes this return. Variations in intrathoracic pressure and cardiac function directly influence intracranial venous flow [38,39]. These effects are particularly pronounced in the jugular veins due to their proximity to cardiopulmonary structures. However, under normal breathing conditions, the percentage of variation in venous flow remains low, as confirmed in subjects breathing normally [35].

Some studies have highlighted other factors influencing vascular flow, such as age [15], posture [5,6], and changes in gravity [15].

### 4.4. Anatomical Studies

Anatomical studies indicate that the diameter of the jugular foramen is generally larger on the right side [40]. This finding is consistent since the right internal jugular vein passes through this foramen and is usually larger on the right. These observations confirm the heterogeneity of the venous system and the tendency for right-sided dominance, consistent with our data (Figure 6).

A potential cause of these anatomical variations may be related to the trajectories of the cranial nerves. The glossopharyngeal and vagus nerves exit the skull through the jugular foramen, and in some cases, the accessory nerve may divide the internal jugular vein [41], which may influence the morphology of the venous system. In a system where a single drainage pathway is used, stenosis of this pathway would have a much more significant impact than in a system with two functional jugular veins.

### 4.5. Venous Drainage and Its Physiological Implications

In this study, we observed that the standard deviations (SDs) are low, ranging from 5% to 9% for cerebral arterial and venous flows at the intracranial level, as well as for cerebral arterial flows measured at the cervical level (Figure 6). These low SD values indicate relative functional homogeneity in these vessels.

However, this pattern changes for veins outside the cranium, where the right and left internal jugular veins present high SDs (21% and 17%, respectively). This reflects significant variability, highlighting the existence of distinct groups. Some individuals demonstrate “right and left jugular dominance” (T1, T2, T3, T6, T10, T12, T13, T20, T22, T23, T25, T26, T27, T31, T32, T34, T35), while others are right jugular dominant (T5, T7, T11, T17, T21, T24, T29, T36), or left jugular dominant (T8, T14, T33). Another group relies on “alternative pathway dominance” for venous drainage (T4, T15, T28) (Figure 7). Finally, a fifth group combines both jugular and the alternative pathway for venous drainage, demonstrating a mixed drainage strategy (T9, T16, T18, T19, T30).

Even though it is well known that cerebral venous drainage is generally predominantly ensured by the internal jugular veins, such heterogeneity in the system, previously highlighted by Alperin et al. [42] and Stoquart-Elsankari et al. [14], raises questions about the origin of these variabilities. The internal jugular veins must adapt to the cranial bony structure at the level of the jugular foramen, which can vary in size [40], potentially influencing their morphology and function.

Intracranially, the venous sinuses primarily drain through the straight sinus and the sagittal sinus, which converge at the confluence of the sinuses. This junction may also present anatomical variations [12].

A study on the effect of internal jugular vein compression in healthy subjects revealed that the “jugular dominant” group experiences an increase in the amplitude of CSF, while the “alternative pathway dominant” group shows a decrease in this amplitude [21]. In cases of jugular vein compression, venous flow decreases, disrupting the balance between arterial inflow and venous outflow. To compensate for this decrease in flow, arterial flow slightly reduces, and the amplitude of CSF increases, likely as a mechanism to maintain stable mean ICP.

In contrast, in the “alternative pathway dominant” group, jugular compression does not produce this effect, as venous drainage remains predominantly posterior, thereby avoiding an increase in CSF amplitude. These findings suggest that anatomical variations and alternative venous drainage pathways influence the venous system’s response to flow disturbances. It seems that, even in a healthy population, all the volunteers are not equal when facing a potential internal jugular compression or blockage. It is interesting to consider which comes first, the pathology or the anatomical variation, as seen in conditions like venous thrombosis [43].

Alterations in the dynamics of the cerebral vascular system can disrupt the regulation of ICP, leading to intracranial hypertension manifested by abnormal elevations in ICP, often accompanied by symptoms such as headaches, nausea, and visual disturbances [44]. Intracranial hypertension can result from various causes, including venous drainage abnormalities, increased intracranial blood volume, or obstruction of CSF.

Other pathologies present symptoms that mimic those of a brain tumor. This is the case with idiopathic cerebral venous thrombosis, which can produce clinical manifestations similar to those of a pseudotumor cerebri, including severe headaches, visual disturbances, and sometimes intracranial hypertension [45]. Cerebral venous thrombosis is a classic example where venous obstruction leads to increased ICP, mimicking the signs of an intracranial tumor.

Arteriovenous fistulas are vascular lesions characterized by abnormal connections between arteries and veins. Unlike arteriovenous malformations, fistulas lack an intermediate “nidus”, which is a capillary network where arterial blood mixes with venous blood. The most common form is the dural arteriovenous fistula, which occurs more frequently in women [46,47]. Although the exact cause of fistulas is still debated, they are thought to often develop after venous sinus thrombosis, possibly related to head trauma [48].

Normal pressure hydrocephalus (NPH) is another condition that can result from altered venous pressure. Disruption in venous drainage can lead to the accumulation of CSF in the cerebral ventricles, causing ventricular dilation without a significant increase in ICP [49]. NPH often manifests a triad of symptoms such as gait disturbances, cognitive impairment, and incontinence [50].

Multiple sclerosis (MS) has also been associated with venous system anomalies, particularly stenosis of the internal jugular veins. This stenosis may play a role in the pathophysiology of MS [51], although subsequent studies have not confirmed significant morphological or venous flow rate differences between MS patients and controls [52,53]. These findings underscore the complexity of the relationship between MS and the cerebral venous system.

Pulsatile tinnitus is another symptom that may be linked to anomalies in the cerebral venous system. Patients often present with morphological anomalies of the sigmoid sinus [54]. This condition, perceived as pulsing sounds synchronized with the heartbeat, can be particularly bothersome and is often associated with turbulence in venous blood flow.

The internal jugular veins are large and easily deformable, while smaller alternative veins have a limited capacity to compensate for redirected flow. This redistribution can lead to an increase in resistance to cerebral venous flow, particularly if the number of veins involved is insufficient. Such changes could potentially affect ICP during an increase in heart rate, such as during physical activity.

**Figure 9:**
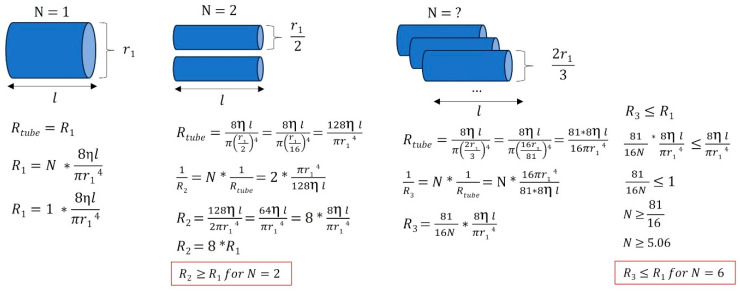
Various venous anatomical configurations and their associated resistances. In this simplified example, we illustrate that when a large jugular vein (with *r* = *r*_1_) is stenosed or absent, smaller vessels draining venous blood can either increase or decrease the equivalent flow resistance, depending on the number of vessels and the radii of the peripheral veins. Different venous tree configurations can result in equivalent cerebral drainage. *R*: resistance, η: viscosity of the fluid considered (here, venous blood), *l*: vessel length, *r*: vessel radius, *N*: number of vessels.

However, vascular resistance not only depends on the caliber of the veins but also on their number. The aim was to understand how different anatomical configurations influence flow resistance compared to a system using a single large internal jugular vein. Assuming the same vessel length and the same fluid viscosity (here, venous blood), Figure 9 shows that flow resistance can either increase or decrease, depending on the number of veins involved.

### 4.6. Pulsatility Index

Extracranial arterial pulsatility shows a significantly higher pulsatility index in men, as shown in Table 3. In contrast, Dai et al. [32] reported no significant sex differences in intracranial venous pulsatility, although their cohort had an older average age (42 ± 13 years) compared to our study.

The reduction in pulsatility is known to play a protective role for the brain by reflecting increased vascular compliance, the capacity of cerebral vessels to buffer pressure fluctuations. Intracranially, the rigid skull confines volume variations. This is partially mitigated by CSF oscillations with the spinal compartment. Along the arterial pathway, the pulsatility index decreases from the common carotid artery to the middle cerebral artery [55], suggesting a progressive attenuation of pulsatility as blood approaches the capillaries, underscoring the compliance of cerebral arteries.

### 4.7. In Pathology

The exploration of venous flow quantification in the internal jugular vein in patients with multiple sclerosis (MS) indicates potential venous dysfunction [56]. However, these observations remain controversial, as similar conditions can also be observed in a healthy population. Criteria for identifying venous failure are based on several observations: low flow in both jugular veins, observed in 3 individuals from our population of 36 (Figure 8); a single dominant jugular vein, identified in 19 individuals from our population (Figure 7); and low pulsatility, with a pulsatility index below 0.3, observed in 8 individuals out of the 68 jugular veins analyzed (Table 3). Regarding the presence or absence of stenosis in our population’s jugular veins, 3 individuals showed no stenosis in either jugular vein, while 5 individuals exhibited unilateral stenosis in one jugular vein.

These findings, although observed in a healthy population, highlight considerable anatomical and functional variability in venous drainage. Such variations could influence intracranial venous pressure regulation and may have clinical implications in pathologies like intracranial hypertension of cerebral venous thrombosis. For instance, in patients with reduced venous outflow capacity or dominant reliance on a single jugular pathway, the system may be more vulnerable to obstruction of pressure imbalances, potentially leading to symptoms such as headache, visual disturbances, or CSF flow disruption. The identification of these venous flow profiles in asymptomatic individuals may help define risk markers or predisposition factors, and could ultimately contribute to improving the diagnosis and management of conditions related to altered venous drainage.

### 4.8. Gravity

Considering the various variations in venous drainage depending on the individual’s posture [5,6], it is interesting to wonder why some individuals in our population exhibit venous drainage patterns similar to those observed in a seated position, even when lying down. Moreover, does this variability persist in a seated position?

In a lying position, blood flows are mainly under the influence of the cardiac pump, which generates a pressure difference between various parts of the venous system. However, transitioning to a standing position activates a compensatory mechanism to counteract the siphon effect and prevent excessive venous pressure. The jugular veins can collapse, meaning they can partially close to redirect venous flow to alternative pathways, often involving smaller-caliber veins. This process allows for effective blood circulation while reducing pressure in the main veins.

This means that, in some individuals, the pattern of blood flow in the veins when lying down resembles what is typically observed in a seated position (Figure 8). This difference could also persist in a seated position, but with specific venous adjustments influenced by gravity and posture.

### 4.9. Limitations and Perspectives

The sample size remains limited, with 36 subjects included, all of whom were healthy and young volunteers. This relatively small cohort may not fully capture the extent of physiological variability across the population but it highlights the fact that cerebral venous anatomy is very variable from one subject to another, and that it could more or less play a role in cases of cerebral pathologies. Future studies should include patients with known venous pathologies to evaluate how these conditions impact venous flow distribution and compensatory mechanisms. Based on the observed variability in venous flow patterns, we estimate that future studies would require more subjects to achieve sufficient statistical power to detect moderate effect sizes. Power calculations were performed using standard assumptions for two-group comparisons, emphasizing the need for larger cohorts to generalize findings.

Additionally, although we calculated the remaining venous flow to infer missing drainage pathways, this approach is indirect and cannot replace direct flow quantification. Some small veins may have gone undetected due to insufficient spatial resolution or small velocities of the flow. Therefore, caution should be exercised in interpreting missing flow signals as anatomical absence.

Another technical limitation lies in the use of our segmentation method providing a fixed ROI across the 32 images of the cardiac cycle. This approach does not account for dynamic changes in vessel position or diameter, potentially affecting flow accuracy even if the change in the vessel area during the cardiac cycle appears to be limited. To better characterize this limitation, future studies could incorporate dynamic segmentation techniques that adapt the ROI to vessel motion across the cardiac cycle, potentially improving the precision of flow measurements. Nevertheless, it was the same method used for all the subjects, minimizing the impact on the comparison made.

**Figure 10:**
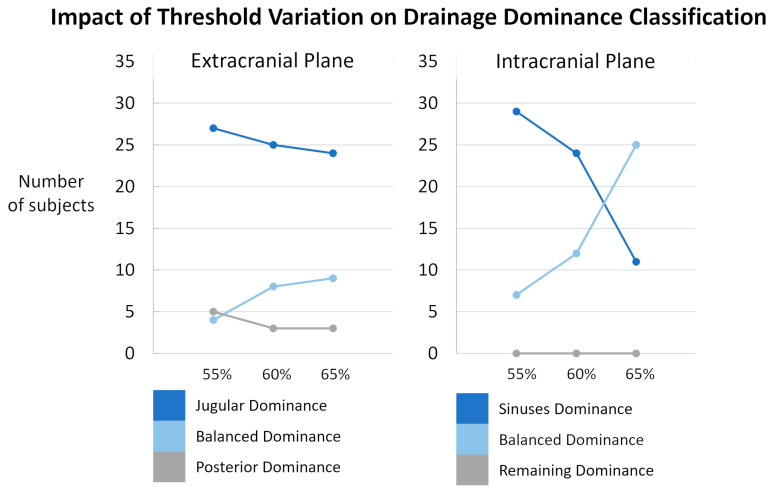
Sensitivity analysis of subject classification according to drainage dominance thresholds. Results are presented separately for the extracranial (**left**) and intracranial (**right**) planes. The proportion of subjects classified as jugular/sinuses dominance (dark blue), balanced dominance (light blue), and posterior/remaining dominance (grey) is shown across different threshold settings: original thresholds (60%), lowered thresholds (55%), and raised thresholds (65%). Minor variations were observed extracranially. Intracranially, the classification appeared more sensitive to threshold variations even though the threshold variations did not affect the dominance classification of the non-sinus drainage.

The threshold used to classify the internal jugular vein and sinus drainage dominance was defined arbitrarily and set at 60% of the total venous flow. A sensitivity analysis was performed by varying this threshold by ±5%, i.e., to 55% and 65% (Figure 10). The impact of threshold showed only minor changes at the extracranial level. The variation in the classification had a little impact on the distribution of subjects among the different dominance categories. At the intracranial level, the classification appeared more sensitive to threshold variations. A marked decrease in sinus dominance was observed when the threshold was changed to 65%. Based on these observations, we chose the 60% threshold as a compromise. Importantly, the threshold variations did not affect the dominance classification of the non-sinus drainage (the “remaining” compartment) at all. This stability can be attributed to the fact that, for the vast majority of the studied population, the sinuses account for more than half of the total venous outflow. Only two quantified subjects exhibited sinus drainage at 48% and 49%, highlighting the sinuses as a major venous drainage pathway. However, the distribution between sinus-dominant and balanced classes was highly sensitive to the selected threshold. In future work, these thresholds could be optimized using data-driven methods or adjusted based on sensitivity analyses to improve the robustness of the classification.

As measurements rely on cardiac gating, respiratory effects are inherently excluded. Previous works using EPI-PC have shown that physiological breathing moderately impacts cerebral arterial flow on the order of 5% [34] and venous flow on the order of 6% [35], and it offers promising avenues for overcoming these limitations in future investigations.

## 5. Conclusions

Our results demonstrate that the quantification of cerebral blood drainage is possible with PC-MRI. Moreover, we identified the presence of venous heterogeneity in the extracranial region, highlighting the complexity and variability of venous networks at the individual level. This approach provides a novel avenue for advancing the understanding of cerebral vascular mechanisms, while offering a promising tool for clinical evaluation of pathologies related to cerebral blood system dysfunction.

## Figures and Tables

**Table 1:** Main parameters of the sequences used. FOV: field-of-view; SENSE: sensitivity encoding; TE: echo time; TR: repetition time.

	3D PC Angiography	2D CINE PC
FOV (mm^2^)	350 × 350	140 × 140
Resolution (mm^2^)	1.5 × 1.5	1 × 1
Thickness (mm)	3	2
Flip angle (°)	12	30
SENSE	-	1.5
TE (ms)/TR (ms)	3/5	7/11
Velocity encoding (cm/s)	30	5–60
Number of images	107	32
Acquisition time min–max (s)	150	40–115
Number of images/cycle	1	32

**Table 2:** Number of quantified vessels and their associated flow rates. Some veins were not present or presented no flow in the extracranial region due to the heterogeneity of cerebral venous drainage. For the posterior veins, there was an average of 3.2 ± 1.3 veins per subject.

Vessels			Number	Mean Flow (mL/min)
Intracranial	Artery	Right internal carotid	36	284 ± 60
		Left internal carotid	36	289 ± 67
		Basilar artery	36	178 ± 61
	Vein	Straight sinus	36	116 ± 37
		Superior sagittal sinus	36	341 ± 61
Extracranial	Artery	Right internal carotid	36	288 ± 47
		Left internal carotid	36	300 ± 47
		Right vertebral	36	99 ± 48
		Left vertebral	36	122 ± 48
	Vein	Right internal jugular	34	329 ± 168
		Left internal jugular	34	207 ± 134
		Right epidural	28	32 ± 39
		Left epidural	27	23 ± 18
		Posterior	116	127 ± 99

**Table 3:** Mean flow and pulsatility index according to slice plane. Student’s *t*-test (*: *p* < 0.05), N (men) = 20; N (women) = 16. ICAs: internal carotid arteries, VAs: vertebral arteries, IJVs: internal jugular veins, EVs: epidural veins, PVs: posterior veins, BA: basilar artery, SS: straight sinus, SSS: superior sagittal sinus.

	Extracranial						Intracranial					
	Arterial (ICAs + VAs)			Venous (IJVs + EVs + PVs)			Arterial (ICAs + BA)			Venous (SS + SSS)		
	Men	Women	*p*	Men	Women	*p*	Men	Women	*p*	Men	Women	*p*
Mean flow (mL/min)	784 ± 80	842 ± 142	0.16	678 ± 90	739 ± 104	0.07	722 ± 145	787 ± 145	0.17	440 ± 94	478 ± 94	0.18
Pulsatility index	1.06 ± 0.25	0.88 ± 0.18	0.02 *	0.67 ± 0.26	0.74 ± 0.26	0.41	0.85 ± 0.14	0.78 ± 0.14	0.23	0.30 ± 0.07	0.29 ± 0.07	0.69

**Table 4:** Mean flow and pulsatility index according to gender. Student’s *t*-test (**: *p* < 0.01, ***: *p* < 0.001).

	Men (N = 20)						Women (N = 16)					
	Arterial			Venous			Arterial			Venous		
	Extra	Intra	*p*	Extra	Intra	*p*	Extra	Intra	*p*	Extra	Intra	*p*
Mean flow (mL/min)	784 ± 80	722 ± 145	0.08	678 ± 90	440 ± 94	2 × 10^−9^ ***	842 ± 142	787 ± 145	0.28	739 ± 104	478 ± 94	2 × 10^−8^ ***
Pulsatility index	1.06 ± 0.25	0.85 ± 0.14	4 × 10^−3^ **	0.67 ± 0.26	0.30 ± 0.07	3 × 10^−6^ ***	0.88 ± 0.18	0.78 ± 0.14	0.09	0.74 ± 0.26	0.29 ± 0.07	2 × 10^−6^ ***

## Data Availability

Due to privacy laws, dicom data cannot be provided. De-identified processed data can be provided upon reasonable request. To obtain the post-processing software and its user manual, please contact livier Balédent at olivier.baldent@chu-amiens.fr.
